# Papulopustular eruptions associated with EGFR-pathway-related targeted therapies: clinical spectrum, evidence boundaries, folliculocentric inflammation, and management considerations

**DOI:** 10.3389/fonc.2026.1864260

**Published:** 2026-07-14

**Authors:** Wei Li, Hui Xu

**Affiliations:** Department of Dermatology, Affiliated Hospital of Jiangsu University, Jiangsu University, Zhenjiang, Jiangsu, China

**Keywords:** Amivantamab, EGFR inhibitors, folliculocentric inflammation, MEK Inhibitors, oncodermatology, papulopustular eruption, skin toxicity, supportive care

## Abstract

Papulopustular eruption is a characteristic dermatologic toxicity of classical epidermal growth factor receptor (EGFR) inhibitors and may impair quality of life, supportive-care needs, and treatment continuity. Although frequently termed acneiform, it is distinct from acne vulgaris and is better understood as a therapy-associated folliculocentric inflammatory eruption. Related papulopustular phenotypes have been reported with MEK inhibitors and newer EGFR-targeting regimens such as amivantamab, but these settings differ in pharmacology, evidence base, and clinical behavior. This Mini Review summarizes these eruptions across three evidence contexts: classical EGFR inhibitor-associated eruption, MEK inhibitor-associated overlapping eruption, and amivantamab-associated scalp-predominant disease in an EGFR/MET bispecific-antibody setting. We discuss mechanisms using an evidence-tiered approach, distinguishing relatively direct evidence for KLF4/IL-36γ signaling and *Cutibacterium acnes*-amplified neutrophilic inflammation from emerging or hypothesis-generating links involving hair-follicle immune dysregulation, JAK-associated signaling, and destructive scalp phenotypes. Finally, we outline evidence-aware management considerations, separating guideline-supported conventional care from lower-level evidence for refractory, superinfected, or scalp-destructive presentations.

## 1 Introduction

Epidermal growth factor receptor (EGFR)-directed therapies are important components of modern cancer treatment, but their clinical use is frequently accompanied by dermatologic adverse events that may impair quality of life, increase supportive-care needs, and threaten treatment continuity (1, 2). Among these toxicities, papulopustular eruption is one of the most characteristic inflammatory cutaneous reactions associated with classical EGFR inhibitors, including anti-EGFR monoclonal antibodies and EGFR tyrosine kinase inhibitors (1). Although the term “acneiform rash” remains widely used, this eruption is clinically distinct from acne vulgaris because comedones are typically absent, lesions arise in close temporal association with systemic anticancer therapy, and other EGFR inhibitor-associated mucocutaneous changes may coexist (3).

As reviewed across molecularly targeted anticancer agents, the relevant clinical landscape is broader than classical EGFR inhibition alone, but it is also heterogeneous (4). Papulopustular eruptions have been reported with MEK inhibitors, supporting phenotypic overlap that is biologically plausible because MEK inhibition disrupts signaling downstream of EGFR and related pathways (5, 6). Newer EGFR-targeting regimens, particularly amivantamab-containing therapy, have drawn attention to scalp-predominant pustular, crusted, erosive, ulcerative, and alopecic presentations in case-series, expert-guidance, and systematic-review literature (7–9). These observations justify comparative discussion, provided that drug-specific pharmacology and evidence boundaries are maintained. Amivantamab is an EGFR/MET bispecific antibody, and its complex scalp-predominant phenotypes may reflect EGFR and MET pathway effects, receptor downmodulation, Fc-mediated activity, combination-regimen effects, secondary infection, or patient-specific scalp vulnerability (10, 11).

Mechanistic studies have moved the field beyond descriptive clinicopathology, but the strength of evidence differs across proposed pathways. EGFR signaling contributes to epidermal differentiation, barrier homeostasis, cutaneous host defense, and hair-follicle biology (12, 13). Experimental evidence supports a keratinocyte-centered inflammatory model in which EGFR/MEK pathway inhibition cooperates with *Cutibacterium acnes*-derived inflammatory signaling to promote KLF4/IL-36γ- and IL-8-associated neutrophilic inflammation (14). Hair-follicle immune dysregulation, including features consistent with immune privilege collapse, may also contribute to chronic, refractory, or scalp-predominant phenotypes in selected settings (15, 16). JAK-linked inflammatory amplification has been implicated in translational models and early intervention studies, but remains less established for destructive scalp disease (17, 18). These mechanisms are therefore best interpreted as a layered and evolving framework rather than as a validated single causal pathway across all agents and presentations.

Supportive-care strategies have shifted from reactive rash treatment toward earlier prevention, phenotype-oriented assessment, and treatment-continuity-focused management (2, 19). However, the evidence base is uneven: conventional EGFR inhibitor-associated papulopustular eruption is supported by guidelines, consensus recommendations, and pre-emptive management studies, whereas refractory, scalp-predominant, and amivantamab-associated complex phenotypes rely more heavily on retrospective studies, case series, systematic reviews, and expert algorithms (8, 20). In this Mini Review, we use “EGFR-pathway-related targeted therapies” as a pragmatic clinicobiologic framework to examine overlapping but non-identical papulopustular and folliculocentric inflammatory eruptions across classical EGFR inhibitors, downstream MEK inhibitors, and amivantamab-containing regimens, with attention to drug-specific pharmacology, evidence strength, and phenotype-specific applicability.

## 2 Clinical spectrum and diagnostic clues

Papulopustular eruption is best established in patients treated with classical EGFR inhibitors, including anti-EGFR monoclonal antibodies and EGFR tyrosine kinase inhibitors (1). The conventional phenotype usually develops early after treatment initiation and predominantly affects seborrheic or folliculosebaceous-rich areas, especially the face, scalp, chest, and upper back (21). Lesions are typically monomorphic inflammatory follicular papules and pustules, often accompanied by erythema, tenderness, burning, pruritus, or xerosis (22). The most practical diagnostic distinction is from acne vulgaris: comedones are usually absent, onset is temporally linked to anticancer therapy, and other EGFR inhibitor-associated mucocutaneous changes such as fissuring or paronychia may coexist (2, 3). Bacterial folliculitis or superinfection should be considered when lesions are unusually painful, purulent, crusted, asymmetric, delayed in onset, or refractory to standard anti-inflammatory and tetracycline-based care (23). Culture and biopsy are not required for typical early seborrheic-site eruptions, but become relevant when lesions are atypical, ulcerative, progressive, treatment-resistant, or associated with suspected scarring alopecia (23, 24).

MEK inhibitor-associated papulopustular eruption represents an overlapping downstream phenotype rather than a pharmacologically identical condition. Early descriptions of selumetinib-associated dermatologic toxicity noted papulopustular rash with pruritus, erythema, and trunk involvement, resembling toxicities seen with EGFR inhibitors (6). Subsequent clinical reviews support this overlap, consistent with disruption of signaling downstream of EGFR and related pathways (5). Combination EGFR inhibitor/MEK inhibitor regimens may further increase severity or broaden distribution, including lower-extremity involvement (25). These observations justify including MEK inhibitor-associated eruption in a comparative EGFR-pathway-related discussion, but they do not establish a single toxicity entity shared by all pathway-targeting agents.

Amivantamab-containing therapy raises a related but distinct diagnostic challenge. As amivantamab is an EGFR/MET bispecific antibody, its dermatologic toxicity should not be interpreted simply as conventional EGFR inhibitor rash in a more severe form (10, 11). Case-series and multicentre data have described conventional papulopustular eruption as well as scalp-predominant pustular, crusted, erosive, ulcerative, and alopecic lesions, including erosive pustular dermatosis-like reactions (7, 26). Expert guidance and systematic review evidence further support separating these complex scalp-predominant presentations from routine early papulopustular eruption (8, 9). Similar destructive scalp inflammation, including folliculitis decalvans-like disease and scarring alopecia, has also been reported with EGFR inhibitors outside the amivantamab setting (27, 28). Therefore, scalp-predominant erosive, crusted, purulent, or alopecic disease should prompt closer dermatologic assessment, lower thresholds for bacterial and fungal cultures, and biopsy when scarring alopecia, ulceration, infection, or an alternative diagnosis is suspected. Existing EGFR inhibitor-specific grading systems remain useful for conventional papulopustular eruption but may not fully capture scalp-destructive or amivantamab-associated phenotypes (24).

## 3 Mechanisms of folliculocentric inflammation under EGFR-pathway-related targeted therapies

Papulopustular eruption under EGFR-pathway-related targeted therapy is best approached mechanistically as a layered model with unequal levels of evidence, rather than as a single validated causal pathway across all agents and phenotypes. At the most established biologic level, EGFR signaling contributes to epidermal differentiation, barrier homeostasis, cutaneous host defense, and hair-follicle biology (12, 13). Disruption of this signaling provides biologic plausibility for folliculocentric inflammation seen most clearly with classical EGFR inhibitors and, more variably, with downstream MEK inhibition.

The most direct mechanistic evidence concerns keratinocyte inflammatory activation under EGFR/MEK pathway inhibition. Satoh and colleagues showed that EGFR inhibitor and MEK inhibitor exposure can cooperate with *Cutibacterium acnes*-derived inflammatory signaling to induce IL-36γ in keratinocytes, followed by IL-8 upregulation and neutrophilic inflammation (14). This response depended on the combined effect of *C. acnes*-mediated NF-κB activation and drug-induced KLF4 upregulation, which together promoted IL36G transcription (14). This model supports KLF4/IL-36γ-associated neutrophilic inflammation as a relatively direct mechanism for acneiform or papulopustular toxicity under EGFR/MEK pathway inhibition. However, it should not be interpreted as proving that all papulopustular, scalp-destructive, or amivantamab-associated presentations share the same driver. In this framework, *C. acnes* is better viewed as a commensal amplifier in a drug-altered epithelial environment than as a universal infectious cause.

A second, emerging level of evidence concerns the hair-follicle immune microenvironment. EGFR inhibitor and MEK inhibitor treatment has been associated with a distinct inflammatory hair-follicle response that includes features consistent with immune privilege collapse (15). This observation is relevant because papulopustular lesions are folliculocentric, and chronic or scalp-predominant disease may involve follicular immune dysregulation beyond conventional epidermal inflammation. A related report also linked EGFR/MEK inhibition to partial hair-follicle immune privilege collapse and excessive IL-33 secretion, suggesting an additional inflammatory pathway in selected settings (16). At present, these findings should be regarded as emerging mechanistic dimensions rather than established universal mechanisms.

A third level consists of translational and hypothesis-generating links involving JAK-associated inflammatory amplification and destructive follicular disease. EGFR deficiency-associated scarring alopecia models have implicated a JAK-STAT1-dependent inflammatory program associated with hair-follicle stem-cell niche exhaustion and tissue destruction (17). Separately, topical JAK inhibition improved EGFR inhibitor-induced rash in preclinical models and early human data (18). These findings support further study of JAK-linked pathways in severe or refractory EGFR-pathway-related cutaneous toxicity, but they do not yet establish JAK inhibition as a standard treatment for scalp-predominant, erosive, or tissue-destructive phenotypes.

Amivantamab requires additional mechanistic caution. Unlike classical EGFR inhibitors, it is an EGFR/MET bispecific antibody that can block ligand binding, promote receptor downmodulation, and engage Fc-mediated immune effector activity (10, 11). Because HGF/MET signaling participates in hair-follicle morphogenesis and cycling, MET pathway perturbation could be biologically relevant to scalp-predominant toxicity (29). Nevertheless, current evidence does not determine whether amivantamab-associated erosive, crusted, pustular, or alopecic scalp disease is driven primarily by stronger EGFR-pathway blockade, MET inhibition, Fc-mediated effects, combination regimens, secondary infection, or patient-specific scalp vulnerability. Therefore, amivantamab-associated scalp-destructive disease should be treated as clinically distinctive but mechanistically unresolved within the broader EGFR-pathway-related toxicity discussion.

**Figure 1:**
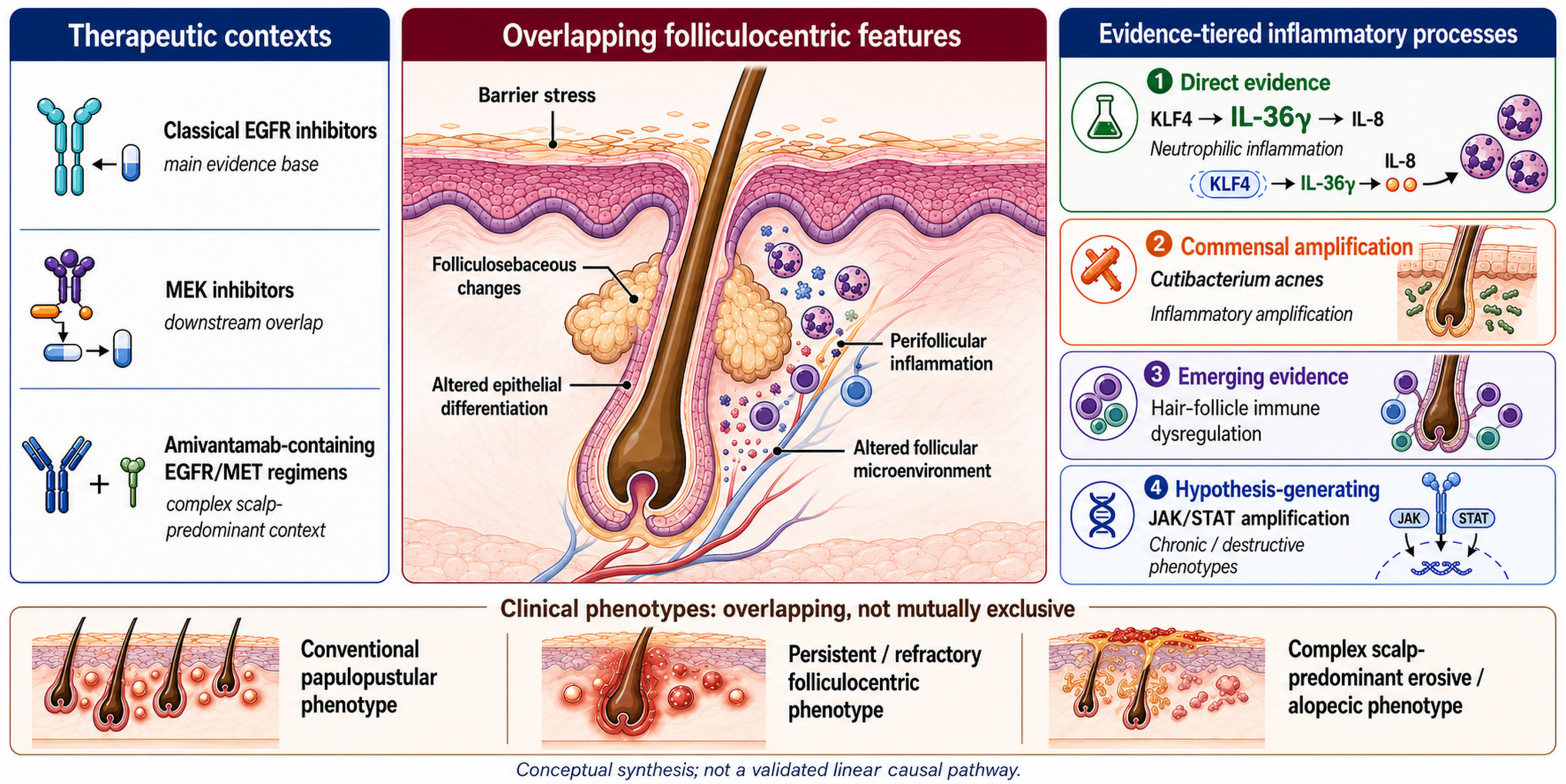
Layered working model of papulopustular eruptions associated with EGFR-pathway-related targeted therapies. The diagram separates three therapeutic contexts: classical EGFR inhibitors, which represent the main evidence base; MEK inhibitors, which show downstream phenotypic overlap; and amivantamab-containing EGFR/MET regimens, which provide a complex scalp-predominant context. Shared folliculocentric features include barrier stress, altered epithelial differentiation, folliculosebaceous changes, perifollicular inflammation, and an altered follicular microenvironment. Evidence-tiered inflammatory processes are shown on the right. The most direct evidence supports KLF4/IL-36γ- and IL-8-associated neutrophilic inflammation under EGFR/MEK pathway inhibition, with *Cutibacterium acnes* acting as a commensal amplifier in a drug-altered epithelial environment. Emerging evidence implicates hair-follicle immune dysregulation, whereas JAK/STAT-associated amplification remains hypothesis-generating, particularly for chronic, refractory, or destructive phenotypes. The lower panel illustrates clinically overlapping phenotypes, including conventional papulopustular eruption, persistent or refractory folliculocentric disease, and complex scalp-predominant erosive or alopecic presentations. This image is intended as a conceptual synthesis and should not be interpreted as a validated linear causal pathway across all agents or clinical phenotypes.

Taken together, the available literature supports a layered working model in which keratinocyte IL-36γ-centered inflammation is the most directly supported component, whereas hair-follicle immune dysregulation, JAK-associated amplification, and drug-specific scalp vulnerability remain less established and phenotype-dependent. This evidence-tiered framework is summarized in **Figure 1**, which separates therapeutic contexts, overlapping folliculocentric features, inflammatory processes with different levels of evidence, and clinically overlapping phenotypes.

## 4 Prevention and management

Management considerations should be interpreted according to agent class, phenotype, severity, infection risk, and oncologic treatment context. For conventional EGFR inhibitor-associated papulopustular eruption, the evidence base is strongest. Clinical practice guidance and prophylactic recommendations support early education, gentle skin care, moisturization, photoprotection, and grade-adapted management of EGFR inhibitor-associated dermatologic toxicities (2, 30). A systematic review also summarized grade-based approaches for EGFR inhibitor-induced skin toxicity (31). The Europe/USA Delphi consensus supports tetracycline-class prophylaxis or early treatment when clinically appropriate, and low-dose isotretinoin may be considered in selected refractory cases (19). A recent systematic review suggested that oral tetracyclines may reduce all-grade or high-grade acneiform rash, while emphasizing the need for further controlled trials (32). Earlier placebo-controlled tetracycline studies produced mixed endpoint-specific results, including benefit for some rash outcomes in one trial and lack of consistent severity reduction in another (33, 34).

Pre-emptive skin-care bundles have prospective support in selected anti-EGFR settings. The STEPP and J-STEPP studies showed that structured pre-emptive skin treatment reduced clinically relevant anti-EGFR skin toxicities during panitumumab-containing therapy (35, 36). Selected topical preventive approaches, such as vitamin K1 cream in cetuximab-treated populations, have also been studied, although these findings should be interpreted within their specific trial contexts (37, 38). For amivantamab-lazertinib, the COCOON trial showed that enhanced dermatologic management reduced grade 2 or higher dermatologic adverse events compared with standard care (20). These data support early, regimen-aware prevention, while also reinforcing that evidence from one drug setting should not be generalized indiscriminately to all EGFR-pathway-related therapies.

Once eruption occurs, treatment should be phenotype- and severity-oriented. Conventional papulopustular eruption is usually managed with continued basic skin care, tetracycline-class therapy, and topical anti-inflammatory treatment according to severity and anatomic site (2, 31). For grade 1–2 facial eruption developing despite pre-emptive care, randomized evidence supports a topical corticosteroid-based approach in the metastatic colorectal cancer setting (39). Most conventional eruptions can be managed while maintaining anticancer therapy, provided symptoms, infection risk, and patient adherence remain acceptable.

Refractory disease requires a more cautious evidence interpretation. Consensus guidance supports dermatology-guided escalation, including low-dose isotretinoin in selected refractory grade II/III cases (19). A retrospective study also suggested that isotretinoin and dapsone may be effective and generally tolerated for treatment-resistant EGFR inhibitor-induced papulopustular exanthema (40). However, these data are observational and should not be interpreted as proving that any escalation strategy improves oncologic outcomes. In practice, refractory conventional eruption should prompt reassessment of diagnosis, adherence, infection, and severity before considering systemic escalation or anticancer dose modification.

Scalp-predominant, erosive, crusted, purulent, ulcerative, or alopecic lesions require separate clinical consideration because the evidence base is lower and the diagnostic stakes are higher. Amivantamab-focused case series, expert algorithms, and systematic reviews describe complex scalp-predominant phenotypes, including erosive pustular dermatosis-like reactions (7–9). Similar folliculitis decalvans-like or scarring alopecia presentations have been reported with EGFR inhibitors outside the amivantamab setting (27, 28). These cases support early dermatology involvement, closer follow-up, and lower thresholds for bacterial and fungal cultures or biopsy when infection, ulceration, scarring alopecia, or an alternative diagnosis is suspected (23). Treatment modification, including dose reduction or temporary interruption, should be individualized through multidisciplinary discussion rather than applied automatically. Patient perspectives, quality-of-life reviews, and real-world surveys indicate that symptom burden, adherence, and practical treatment burden can affect skin-toxicity management and treatment continuity (41–43). These phenotypes should be interpreted as overlapping rather than mutually exclusive; for example, refractory or scalp-predominant disease may coexist with suspected superinfection. **Table 1** provides a narrative synthesis of phenotype-oriented clinical considerations and evidence boundaries rather than a validated management pathway.

**Table 1:** Phenotype-oriented clinical considerations and evidence boundaries for papulopustular eruptions associated with EGFR-pathway-related targeted therapies.

Phenotype/context	Key clues	Evaluation triggers	Clinical implications	Evidence basis and boundary
Conventional EGFR inhibitor-associated papulopustular eruption	Early onset; seborrheic or folliculosebaceous distribution; monomorphic papules and pustules; absence of comedones; burning, pruritus, tenderness, or xerosis	Usually clinical diagnosis; culture or biopsy generally unnecessary in typical early disease	Gentle skin care, moisturization, photoprotection, tetracycline-class prophylaxis or early treatment may be considered according to risk and local practice; anticancer therapy can often be maintained when clinically appropriate	Best-supported phenotype. Evidence includes guidelines, consensus recommendations, systematic reviews, and randomized or prospective pre-emptive management studies in selected EGFR inhibitor settings (2, 19, 35). Tetracycline prophylaxis evidence is supportive but not fully uniform (32–34).
MEK inhibitor-associated overlapping papulopustular phenotype	Papulopustular rash with pruritus, erythema, trunk involvement, or broader distribution; may resemble EGFR inhibitor-associated eruption	Consider alternative diagnoses if distribution, timing, or morphology is atypical	Similar supportive and anti-inflammatory principles may be considered, but interpretation should reflect the specific MEK inhibitor, combination regimen, and oncology context	Evidence supports phenotypic overlap, not pharmacologic equivalence. Data are mainly from clinical descriptions, reviews, and combination-therapy series (5, 6, 25).
Refractory inflammatory phenotype	Persistent or progressive eruption despite standard care; broader inflammatory burden; treatment intolerance or adherence difficulty	Reassess diagnosis, adherence, severity, infection, and concomitant therapies; consider culture or biopsy if delayed, painful, purulent, asymmetric, or treatment-resistant	Dermatology-guided escalation may be considered; low-dose isotretinoin or dapsone has been reported in selected cases; anticancer dose modification is individualized	Evidence includes consensus guidance and retrospective data. Escalation strategies are not validated as universal algorithms and do not prove oncologic benefit (19, 40).
Suspected superinfected phenotype	Purulence, malodor, marked pain, crusting, asymmetry, unexpected worsening, or poor response to anti-inflammatory therapy	Bacterial culture is reasonable when infection is suspected; fungal evaluation or biopsy may be considered when diagnosis remains uncertain	Supportive care can continue while infection is evaluated; antimicrobial treatment should follow culture results and clinical context; avoid escalating immunosuppression before infection is considered	Evidence supports diagnostic vigilance rather than automatic attribution to inflammatory rash alone. This is a diagnostic consideration rather than a validated treatment pathway (23).
Complex scalp-predominant phenotype, including amivantamab-associated presentations	Scalp pustules, erosions, crusting, ulceration, alopecia, suspected scarring, or erosive pustular dermatosis-like lesions	Early dermatology assessment; lower threshold for bacterial and fungal cultures; biopsy when scarring alopecia, ulceration, infection, or alternative diagnosis is suspected	Phenotype-specific scalp care and closer follow-up are reasonable; escalation of dermatologic care or anticancer dose modification may be considered through multidisciplinary discussion when disease is progressive, destructive, infected, or intolerable	Lower-level but clinically important evidence. Data include amivantamab case series, expert algorithms, and systematic review evidence (7–9). EGFR inhibitor-associated scarring alopecia and folliculitis decalvans-like presentations are supported mainly by case-series evidence (27, 28). COCOON supports enhanced dermatologic management for amivantamab-lazertinib, but destructive scalp phenotypes remain mainly supported by case-based and expert evidence (20).

EGFR, epidermal growth factor receptor. This table is intended as a narrative synthesis of evidence-aware clinical considerations, not as a validated management algorithm. Specific supporting references are provided in the main text. Existing EGFR inhibitor-specific grading systems remain useful for conventional papulopustular eruption but may not fully capture scalp-destructive or amivantamab-associated phenotypes (24).

## 5 Discussion and future directions

Papulopustular eruptions associated with EGFR-pathway-related targeted therapies are best understood as overlapping but non-identical folliculocentric inflammatory toxicities. The strongest clinical and management evidence remains in the setting of classical EGFR inhibitors, where the conventional early papulopustular phenotype, diagnostic distinction from acne vulgaris, and supportive-care strategies are relatively well established (1, 19). MEK inhibitor-associated eruptions support downstream pathway-related overlap, but do not by themselves establish pharmacologic equivalence with EGFR inhibitor-induced rash (5, 6). Amivantamab-containing regimens further broaden the clinical discussion by introducing complex scalp-predominant erosive, crusted, pustular, and alopecic presentations in an EGFR/MET bispecific-antibody context (7, 8).

Several limitations should be emphasized. This Mini Review is narrative rather than systematic and does not provide quantitative estimates of incidence, risk factors, or treatment effects. The EGFR-pathway-related framework is used to organize overlapping clinical and biologic observations while preserving distinctions among classical EGFR inhibitors, MEK inhibitors, and amivantamab-containing regimens. Mechanistic evidence also varies in strength: IL-36γ-centered neutrophilic inflammation is supported by relatively direct experimental data (14). Hair-follicle immune dysregulation and IL-33-associated findings remain emerging mechanisms (15, 16). JAK-associated signaling remains translational or hypothesis-generating in this context (17, 18), whereas amivantamab-associated scalp-destructive disease remains clinically distinctive but mechanistically unresolved, supported mainly by case-based and expert literature (7, 8). Accordingly, **Table 1** should be read as a narrative evidence synthesis rather than a validated clinical algorithm.

Several gaps require specific attention. Terminology remains inconsistent across studies, and existing EGFR inhibitor-specific grading systems are most useful for conventional papulopustular eruption but less suited to scalp-destructive or amivantamab-associated phenotypes (24). Prospective studies should therefore use agent-specific and phenotype-stratified definitions, separating conventional early seborrheic-site eruption from refractory inflammatory disease, suspected superinfection, and scalp-predominant erosive or alopecic presentations. Biomarkers predicting severity, chronicity, treatment resistance, or scarring risk are also lacking. Candidate clinical, histologic, inflammatory, and microbiologic markers require prospective validation, particularly for scalp-predominant, refractory, or scarring phenotypes (14, 17, 23).

Future supportive-care research should move from broad rash control toward phenotype-stratified and regimen-specific validation. Conventional EGFR inhibitor-associated papulopustular eruption can continue to be studied through preventive bundles, tetracycline-based strategies, and topical anti-inflammatory approaches. Refractory disease requires prospective evaluation of escalation strategies such as isotretinoin or dapsone, with careful separation of dermatologic improvement from oncologic outcomes (40). Scalp-predominant destructive disease, particularly in amivantamab-containing regimens, requires separate studies addressing diagnostic work-up, infection assessment, scarring risk, and criteria for systemic escalation or treatment interruption (20). Mechanism-directed approaches, including topical blockade of drug-receptor interactions or JAK-linked interventions, are promising but require phenotype-specific validation before routine use (18, 44).

In summary, papulopustular eruptions under EGFR-pathway-related targeted therapies provide a useful model for integrating clinical phenotyping, epithelial and follicular immunobiology, and supportive-care innovation. The most productive next step is not to assume a unified mechanism or universal management pathway, but to define which mechanisms, risk markers, and interventions apply to specific agents, phenotypes, and treatment contexts.
